# Bioinformatics-guided construction of a tumor microenvironment-derived prognostic model in acute myeloid leukemia

**DOI:** 10.1371/journal.pone.0325145

**Published:** 2025-07-03

**Authors:** Amir Abbas Navidinia, Reza Khayami, Alireza Gholami, Mahnaz Fathi, Ali Keshavarz, Najibe Karami, Hirad Alipanah, Ali Ahmadi, Shahrbano Rostami, Bahram Chahardouli

**Affiliations:** 1 Hematology-Oncology and Stem Cell Transplantation Research Center, Tehran University of Medical Sciences, Tehran, Iran; 2 Department of Medical Genetics and Molecular Medicine, Faculty of Medicine, Mashhad University of Medical Sciences, Mashhad, Iran; 3 Faculty of Medicine, Student Research Committee, Hormozgan University of Medical Sciences, Bandar Abbas, Iran; 4 Department of Hematology, Faculty of Medical Sciences, Tarbiat Modares University, Tehran, Iran; 5 Department of Hematology and Blood Banking, School of Allied Medical Sciences, Shahid Beheshti University of Medical Sciences, Tehran, Iran; 6 PhD Student at Electrical and Computer Engineering, University of Pittsburgh, Pittsburgh, Pennsylvania, United States of America; 7 Hematologic Malignancies Research Center, Research Institute for Oncology, Hematology and Cell Therapy, Shariati Hospital Tehran University of Medical Sciences, Tehran, Iran; 8 Cell Therapy and Hematopoietic Stem Cell Transplantation Research Center, Research Institute for Oncology, Hematology and Cell Therapy, Tehran University of Medical Sciences Tehran, Tehran, Iran; Xiangya Hospital Central South University, CHINA

## Abstract

**Background:**

The tumor microenvironment (TME) exerts a profound influence on the progression, therapeutic responses, and clinical outcomes of acute myeloid leukemia (AML), a prevalent hematologic malignancy in adults. This study aimed to establish a TME-based prognostic model to unveil novel therapeutic and prognostic avenues for AML.

**Methods:**

Gene expression profiles and clinical information for 134 AML patients were retrieved from The Cancer Genome Atlas (TCGA). The TME cellular components were evaluated using the ESTIMATE algorithm, and differentially expressed genes (DEGs) were identified. A Microenvironment Prognostic Model (MPM) was subsequently constructed through univariate Cox regression, LASSO regression, and multivariate Cox regression analyses. The predictive performance of the MPM was validated in a separate cohort of 312 AML patients from the TARGET database.

**Results:**

Kaplan-Meier analysis revealed significant associations between the TME, French-American-British (FAB) classification, and overall survival (*p-values* = 3.6e-07 and 0.011, respectively). LASSO-Cox regression identified eight essential genes (CXCL12, GZMB, ITPR2, LYN, RAB9B, RGMB, RUFY4, TRIM16) that exhibited a strong correlation with survival (*p-value* < 0.0001). The MPM demonstrated excellent prognostic performance, with area under the curve (AUC) values of 84.05, 85.73, and 89.54 for predicting 1-, 3-, and 5-year survival, respectively. External validation with the TARGET database underscored the robustness of this model, yielding AUC values of 60.5%, 56.7%, and 55.7% at the corresponding intervals.

**Conclusion:**

These findings present a TME-based prognostic model that offers a promising avenue for precise risk stratification and targeted therapeutic strategies in AML.

## 1 Background

Acute myeloid leukemia (AML), the most prevalent form of adult acute leukemia, arises from the unchecked proliferation of myeloid precursor cells. This abnormal growth disrupts normal blood cell production, leading to bone marrow failure [1,2]. Despite achieving complete remission with initial induction chemotherapy, AML patients face a disappointingly low five-year survival rate, primarily due to frequent relapses. These relapses are often attributed to minimal residual disease (MRD) ensconced within a protective tumor microenvironment (TME) that promotes immune evasion and resistance to treatment [3,4].

The tumor microenvironment (TME) constitutes a spatially organized, metabolically dynamic niche where malignant cells co-opt stromal fibroblasts, endothelial cells, immunosuppressive myeloid populations, and extracellular matrix (ECM) components to drive tumor progression and therapeutic resistance [5–8]. AML blasts actively remodel their niche through complex interactions with mesenchymal stromal cells, endothelial cells, and immunosuppressive myeloid populations, which together establish a cytokine- and chemokine-rich milieu [9–11]. This remodeled niche supports leukemic stem cells (LSCs) through CXCR4/CXCL12-mediated retention, CD44/VLA-4 adhesion, and survival signals (VEGF, TGF-β) while suppressing normal hematopoiesis [12]. The TME confers chemoresistance via hypoxic sanctuaries, mitochondrial transfer, and NF-κB/STAT3 activation [13]. Therapeutic targeting remains challenging due to niche plasticity and hematopoietic toxicity, though emerging approaches combining CXCR4 inhibitors (plerixafor) with chemotherapy or metabolic disruptors show promise [14,15]. Thus, a comprehensive understanding of these dynamic microenvironmental interactions is therefore critical for developing more effective therapeutic strategies to overcome treatment resistance and prevent disease relapse.

Rapid strides in microarray and next-generation sequencing (NGS) technologies now enable precise prognostication and customization of treatment for AML patients [16]. The ESTIMATE algorithm stands out by effectively quantifying immune and stromal cell infiltration within the TME, and has been employed across various cancers including those of the gastric cancer [17], breast cancer [18], prostate cancer [19], colon cancer [20], osteosarcoma [21], renal cell carcinoma [22] and hepatocellular carcinoma [23]. This approach has also facilitated the determination of immune and stromal scores specifically for AML patients [24–28].

Despite extensive research into novel therapeutic avenues and drugs, the relapse and mortality rates in AML remain stubbornly high. Accurately predicting patient outcomes at diagnosis is therefore crucial [29]. Existing prognostic models, which incorporate factors like leukemia hematopoietic stem cells (LSC), microRNAs, gene expression patterns, methylation profiles, and markers of immunogenic cell death, often show limitations, particularly in their effectiveness across different AML subtypes [30–35]. Consequently, there is an urgent and pressing need for the development of more refined prognostic models. To address these challenges, our research introduces an innovative prognostic model that integrates gene expression data from AML patient cohorts in both the Cancer Genome Atlas (TCGA) and the Therapeutically Applicable Research to Generate Effective Treatments (TARGET) databases, refined further with the ESTIMATE algorithm to boost its predictive precision.

## 2 Methods

### 2.1 Data requisition

The level 3 RNA sequencing data with corresponding clinical information of 151 newly diagnosed AML patients from the TCGA database and 312 AML patients from the TARGET database were downloaded from the GDC database (https://portal.gdc.cancer.gov/repository). The patient data derived from the TCGA database were employed in the construction of the prognostic model, while the data from the TARGET database were applied for the external validation of the model. Within this patient of TCGA cohort, 17 individuals were identified as having incomplete clinical records, notably in relation to their survival data. In line with our principal aim of developing a prognostic model, which hinged on the availability of comprehensive clinical details, these 17 patients were subsequently omitted from the analytical process, resulting in a final cohort of 134 patients for further analyses.

### 2.2 Microenvironment-related differentially expressed genes

In order to assess the quality of stromal and immune cells in the TME of the patients with AML, an ESTIMATE analysis was performed. According to the median of their ESTIMATE scores, AML patients were categorized into low and high groups. The “DESeq2” package was utilized to obtain differentially expressed genes (DEGs) between high and low ESTIMATE groups. Genes with a |Fold Change| higher than 1.5 and a false discovery rate (FDR) lower than 0.05 were considered DEGs. The “pheatmap,” “plotPCA,” and “ggplot2” packages were utilized to perform heatmap, PCA, and volcano plots, respectively.

### 2.3 Gene ontology and KEGG pathway

The DEGs were analyzed using DAVID (http://david.niaid.nih.gov) for Gene Ontology (GO), REACTOME, and Kyoto Encyclopedia of Genes and Genomes (KEGG), with statistical significance at P < 0.05.

### 2.4 Protein-protein interaction analysis

STRING version 12 (https://string-db.org/) was utilized to investigate interactions between DEGs using Protein-Protein Interaction (PPI) analysis. The database settings were configured with a required score set to medium confidence and a False Discovery Rate (FDR) stringency of 5%. The results of this PPI analysis were subsequently imported into Cytoscape v.3.10.1 to enable the construction of a network model, providing insights into the intricate interplay among these DEGs. The top ten hub DEGs Were identified using Cytohubba, a plug‑in for Cytoscape, for closeness, betweenness, and degree algorithms for both upregulated and downregulated DEGs.

### 2.5 Survival analysis and prognostic model construction

To construct a Microenvironment-Prognostic Model (MPM), the initial step involved the execution of univariate Cox regression analysis with the R package “survival” (Version 3.8–3) to determine the associations between DEG expression levels and overall patient survival. DEGs with a significance level of P < 0.05 in univariate Cox regression were identified as predictive genes. Subsequently, the dataset was randomly partitioned into training and test groups to validate the model’s accuracy. The train set was utilized to construct MPM, while the testing set and the entire dataset were utilized to validate the prediction signature. The “glmnet” package (version 4.1–8) was used to perform Least absolute shrinkage and selection operator (LASSO) regression analysis (with a penalty parameter determined by 10-fold cross-validation) to narrow the risk of overfitting. Multivariate Cox regression analysis was used to generate the risk score (RS) for each AML patient, which is statistically equivalent to Σ (βi * Expi) (i = the number of prognostic hub genes).

To assess the model’s accuracy comprehensively, an array of R packages, including “survival”, “caret,” “glmnet”, “rms”, “survminer”, and “timeROC” were employed. These packages facilitated the execution of various analyses, including Kaplan-Meier analysis and the generation of receiver operating characteristic (ROC) curves for 1, 3, and 5-year survival across the training, testing, and entire patient datasets. Additionally, the calculation of the area under the curve (AUC) was carried out, providing a valuable measure of the model’s predictive performance for training, testing, and entire patient datasets. A higher AUC value indicated enhanced predictability of the Microenvironment-Prognostic Model (MPM) under the ROC curve.

To assess the predictive capacity of the MPM in comparison to the ESTIMATE algorithm and age, ROC curve analysis was carried out across 1, 3, and 5-year intervals. Furthermore, external validation of the MPM was performed using AML patient data from the TARGET database, involving Kaplan-Meier analysis and ROC curves for 1, 3, and 5-year predictions to affirm the model’s predictive robustness. Subsequently, a nomogram model was established for forecasting survival years in AML patients by incorporating the risk score and various clinical features, such as age, FAB classification, and Cancer and Leukemia Group B (CALGB) stage, utilizing the “rms” and “survival” packages. The Consistency Index (C-index) was then computed to assess the model’s accuracy and provide insights into its reliability and effectiveness. Additionally, a comprehensive examination was conducted to explore the correlations between the MPM and various clinical factors, encompassing variables like age, FAB classification, and CALGB stage.

### 2.6 Statistical analysis

All statistical analyses were performed in R software (version 4.4.2; Auckland, New Zealand, United States). The Kruskal–Wallis test was applied to evaluate differences among multiple groups, acknowledging the non-parametric nature of the data. For two-group comparisons, the Wilcoxon rank-sum test was employed. Statistical significance was defined as P < 0.05.

## 3 Results

### 3.1 ESTIMATE scores are associated with AML clinical parameters

After excluding 17 AML patients with incomplete clinical information, 134 patients remained (**Table 1**). Of these patients, 76 (56.71%) were male, and 58 (43.28%) were female. The median age at initial pathological diagnosis was 58 years, ranging from 21 to 88 years. The fourteen subtypes of these patients were M0 undifferentiated (14, 10.6%), M1 (30, 22.7%), M2 (32, 24.2%), M3 (14, 10.6%), M4 (27, 20.5%), M5 (12, 9.1%), M6 (2, 1.5%), and M7 (1, 0.8%); two patients were not classified. Subsequently, we determined the ESTIMATE scores for each patient using the ESTIMATE algorithm.

**Table 1:** Clinical characteristics of the TCGA AML cohort.

	Number/ range	Percentage (%)
**Sex**		
Male	76	56.72
Female	58	43.28
**FAB classification**		
M0	14	10.45
M1	30	22.39
M2	32	23.88
M3	14	10.45
M4	27	20.15
M5	12	8.96
M6	2	1.49
M7	1	0.75
Not classified	2	1.49
**CALGB category**		
Favorable	29	21.64
Intermediate	76	56.72
Poor	27	20.15
NA	2	1.49
**Age**		
< 60 years	73	54.48
> 60 years	61	45.52
**Continuous variables**	**Range**	**Median**
Stromal Score	−1582.2 - 425.9	−937.4
Immune Score	1243–3669	2462
ESTIMATE Score	−218.8 - 4094.9	1500.7
Age	21–88	58

**Fig 1:**
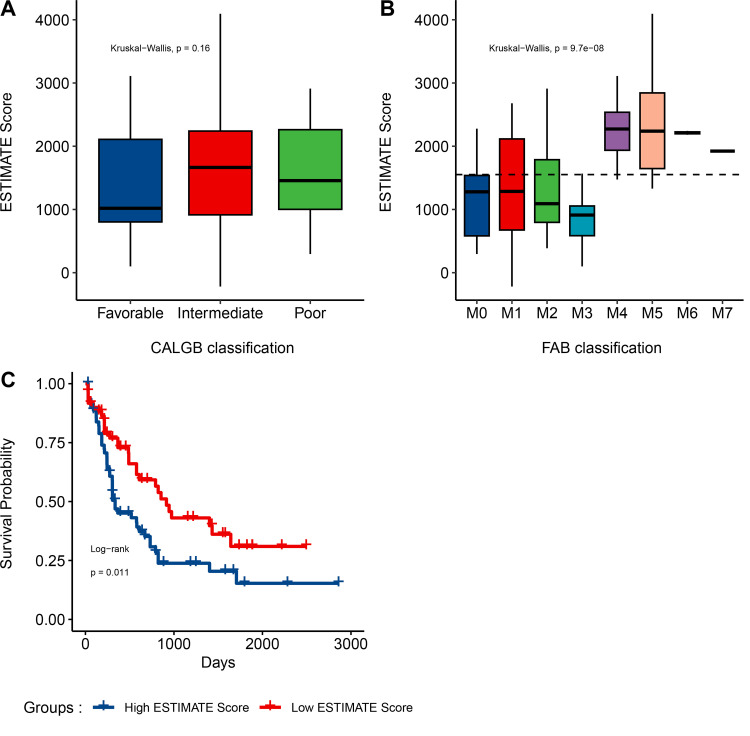
Association of ESTIMATE scores with AML clinical features. **A**, The correlation between ESTIMATE scores and AML cytogenetic risk (*P* = 0.16). **B**, Distribution of ESTIMATE scores for AML subtypes (*p-value* = 9.7e-08). **C**, Kaplan-Meier survival curve reveals that higher ESTIMATE scores are associated with significantly shorter overall survival (log-rank test, *p-value* = 0.011).

In order to evaluate the association of ESTIMATE scores with AML cytogenetic risk, we classified the cytogenetic risk of AML patients as favorable, intermediate/normal, or poor and plotted the distribution of ESTIMATE scores concerning the level of cytogenetic risk; however, the result was not significant (*p-value* = 0.16; **Fig 1-A**). On the other hand, ESTIMATE scores were significantly associated with the FAB classification (*p-value* = 9.7e-08; **Fig 1-B**). Moreover, the AML patients were divided into high- and low-score groups to investigate the potential relationship between ESTIMATE scores and overall survival. Patients with low ESTIMATE scores had a longer median overall survival than those with high ESTIMATE scores (*p-value* = 0.011; **Fig 1-C**).

### 3.2 Identification of differentially expressed genes (DEGs) based on Estimate scores in AML

**Fig 2:**
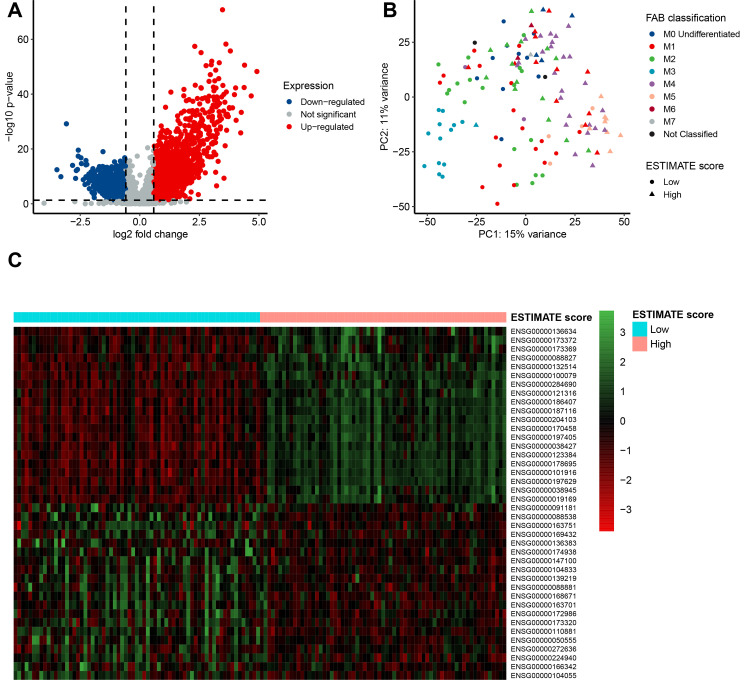
Identification of DEGs based on ESTIMATE scores. **A,** Volcano plot of DEGs from the low vs. high stromal score groups. Genes with p < 0.05 are shown in red (fold change > 1.5) and blue (fold change <−1.5). Grey plots represent the remaining genes (those with no significant difference). **B,** PCA plot of TCGA data based on ESTIMATE scores and FAB classification. **C,** Heatmap of top-20 upregulated-DEGs and top-20 downregulated-DEGs for the ESTIMATE score groups.

We evaluated the RNA-Seq data of the patients to examine the relationship between gene expression profiles and ESTIMATE scores. Using the cut-off criteria of *p-value* = 0.05 and |log2 fold change| > 1.5, 2134 DEGs (1380 commonly upregulated genes and 754 commonly downregulated genes) were found based on ESTIMATE scores (**Fig 2-A**). Moreover, the principal component analysis (PCA) was performed to assess the relation between ESTIMATE scores and FAB classification (**Fig 2-B**). The DEGs of the low versus high ESTIMATE score groups are depicted in Fig 2-C’s heatmap (**Fig 2-C**). The focus of our subsequent analysis was on these common DEGs.

### 3.3 Gene ontology

**Fig 3:**
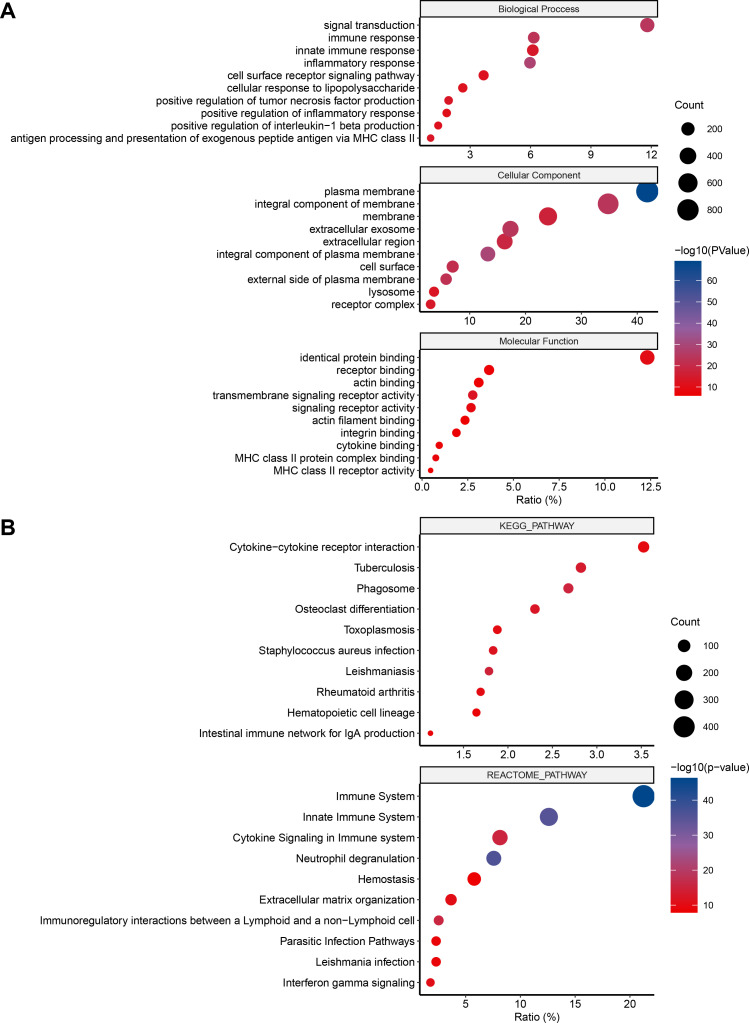
GO term enrichment analysis of common DEGs. **A,** the top 30 significantly enriched GO terms, including three sub-ontologies, biological process, molecular function, and cellular component, are shown. **B,** Interrelation analysis of KEGG and REACTOME pathways of common DEGs.

Gene ontology (GO), KEGG, and REACTOME pathway analyses were used to investigate the biological processes and pathways involved. Using the DAVID gene annotation tool, the DEGs were analyzed for three sub-ontologies, as shown in **Fig 3-A**: biological processes (BP), cellular components (CC), and molecular function (MF). Regarding BP, DEGs were most enriched in neutrophil degranulation, inflammatory response, immune response, signal transduction, and cytokine-mediated signaling pathways. KEGG pathway enrichment and interrelationship showed that the DEGs involved the cytokine-cytokine receptor interaction, phagosome, tuberculosis, and osteoclast differentiation (**Fig 3-B**). REACTOME pathway analysis revealed that the top pathways related to DEGs were the immune System, neutrophil degranulation, innate immune System, immunoregulatory interactions between a lymphoid and a non-lymphoid cell, toll-like receptor cascades, and cytokine signaling in the immune system (**Fig 3-C**).

### 3.4 Protein-protein interaction (PPI) network construction and functional enrichment of genes of prognostic value

**Fig 4:**
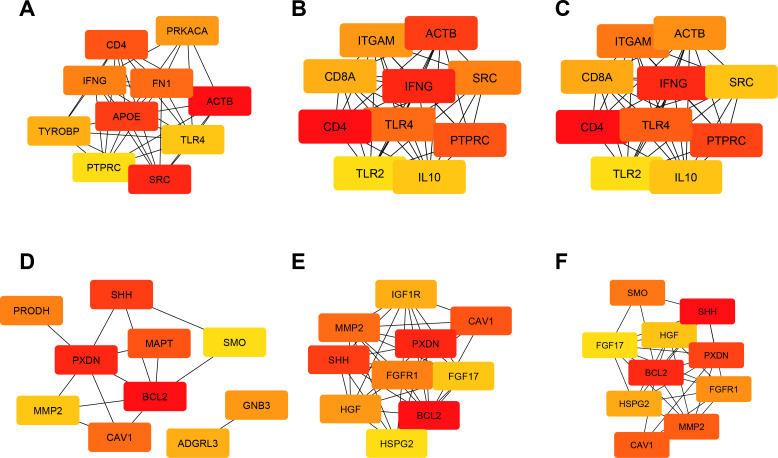
The PPI network consists of the top 10 hub upregulated and downregulated DEGs according to the cytoHubba analysis. The algorithms are: **A,** betweenness of top 10 upregulated-DEGs; **B,** closeness of top 10 upregulated-DEGs; **C,** Degree of top 10 upregulated-DEGs. **D,** betweenness of top 10 downregulated-DEGs; **E,** closeness of top 10 downregulated-DEGs; **F,** Degree of top 10 downregulated-DEGs. The red indicates a higher score, and the yellow indicates a lower score.

We made a PPI network using the STRING online database and Cytoscape software to investigate the interactions between upregulated and downregulated DEGs. The supplementary shows that the network of upregulated DEGs contains 1366 nodes and 17147 edges, and the network of downregulated DEGs contains 739 nodes and 1238 edges. The STRING data were then further analyzed using Cytoscape, and closeness, betweenness, and degree were identified for upregulated DEGs (**Fig 4A-C**) and downregulated DEGs (**Fig 4D-F**) using cytoHubba.

### 3.5 Microenvironment prognostic model establishment

**Fig 5:**
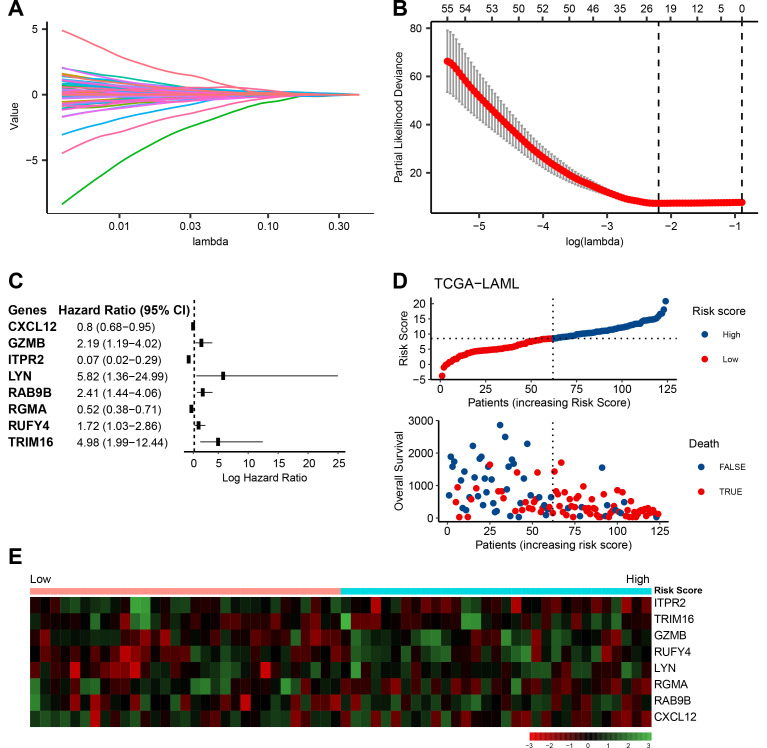
Establishment of MPM. **A,** LASSO coefficient profiles of the prognostic DEGs. **B,** Ten-fold cross-validation for tuning parameter selection in the LASSO model. The partial likelihood deviance is plotted against log (λ), where λ is the tuning parameter. Partial likelihood deviance values are shown, with error bars representing SE. The dotted vertical lines are drawn at the optimal values by minimum criteria and 1-SE criteria. **C,** Forest plot of hazard ratios for 8 prognostic DEGs. **D,** Distributions of risk score and overall survival status according to risk score increment. **E,** Expression profile of signature genes in high and low risk score groups.

In order to construct a microenvironment prognostic model (MPM), initially, we performed a univariate Cox regression analysis on the DEGs. Of 2134 microenvironment-related genes, 733 were prognostic. The LASSO regression was performed to avoid overfitting, and 24 genes were selected for further analysis. The multi-cox proportional hazard test revealed that eight genes were strongly associated with the overall survival of AML patients (**Figs 5A-C**). The expression levels of these eight genes and their respective coefficients derived from the multi-Cox proportional hazard test were used to calculate individual-level risk scores for each patient. The following formula was used for calculating each patient’s risk score: risk score = ITPR2 × (−2.695558) + LYN × (1.762128) + RGMA × (−0.657528) + GZMB × (0.783182) + RAB9B × (0.880839) + CXCL12 × (−0.219737) + RUFY4 × (0.540176) + TRIM16 × (1.605168). Examination of the risk factors linked to these eight genes revealed a positive relation between increased gene expression and a heightened risk of mortality (**Fig 5D**, **5E**).

**Fig 6:**
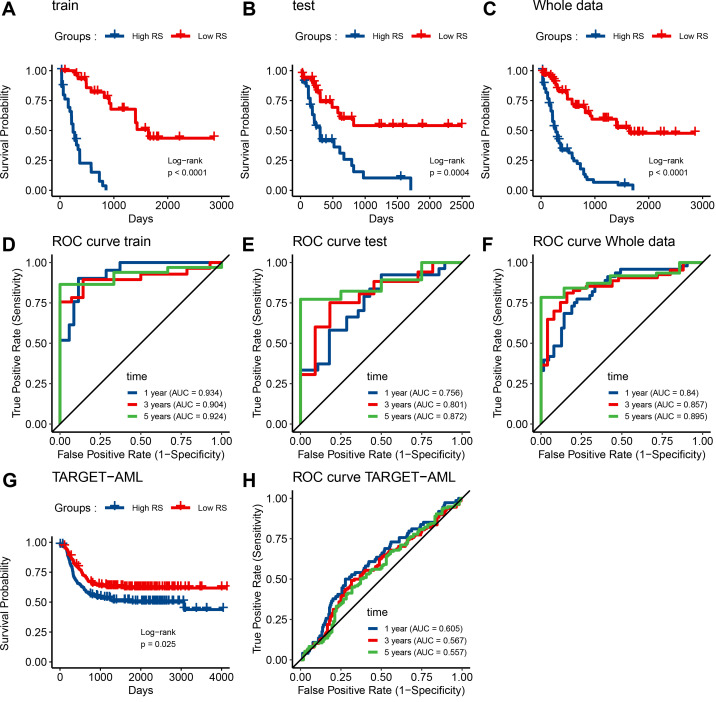
Evaluating the Prognostic Efficacy of MPM in AML. **A-C**, Kaplan–Meier analysis substantiates the robust prognostic relevance of MPM within the training, test, and overall patient cohorts, exhibiting statistical significance (*p-values* <0.0001, = 0.0004, < 0.0001, respectively). **D-F,** Time-dependent ROC curves elucidate the precision of MPM in forecasting 1-, 3-, and 5-year Overall Survival rates among patients within the TCGA dataset. **G,** External validation using TARGET data corroborates MPM’s significant relationship with AML prognosis. **H,** Time-dependent ROC curves further highlight the MPM’s competence in predicting 1-, 3-, and 5-year OS rates within the TARGET AML patient population.

We obtained the Risk Score for all patients and then classified them as low or high risk based on the median. A Kaplan-Meier survival analysis of test, train, and whole data showed that the high-risk group had a considerably lower survival rate than the low-risk group (*p-value* < 0.0001, *p-value* = 0.00041, and *p-value* < 0.0001, respectively; **Fig 6A-C**). The ROC curve was constructed to test the model’s accuracy in test, train, and entire data (**Fig 6D-F**). Especially, the AUC of 1, 3, and 5-year survival for entire data were 84.05%, 85.73%, and 89.54%, respectively, which indicate the robust predictive power of our prognostic model across different timeframes.

Crucially, it is worth emphasizing that our prognostic model demonstrated superior predictive accuracy compared to both the ESTIMATE algorithm and age. When evaluating ROC curves for 1, 3, and 5-year survival, the AUC values for the ESTIMATE algorithm were 64.6%, 63.2%, and 71.2%, respectively, while age yielded AUC values of 68.8%, 72.8%, and 79.3%. This contrast underscores the enhanced predictive capability of our model in foreseeing AML patient outcomes (**S1A**, **S1B Fig**).

Furthermore, we applied TARGET data comprising 312 AML patients to conduct external validation of the prognostic model. Our analysis, which included Kaplan-Meier survival curves, revealed a statistically significant relationship between the risk score and patient survival within this dataset (*p-value* = 0.025; **Fig 6G**, **6H**). Additionally, we generated ROC curves to assess the model’s validity across 1, 3, and 5-year intervals, yielding respective AUC values of 60.5%, 56.7%, and 55.7%, respectively.

### 3.6 Nomogram model construction

**Fig 7:**
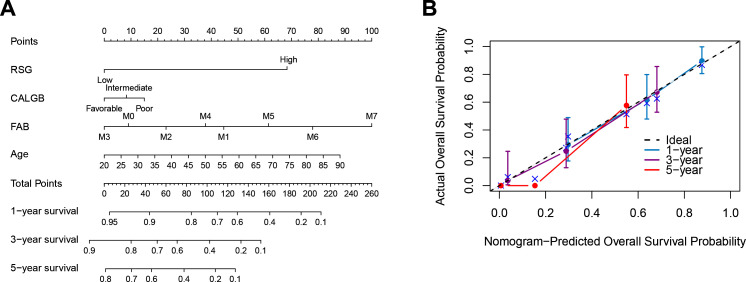
Nomogram Development for Survival Prediction in AML. **A,** Nomogram displaying the predictive factors, including RSG, age, FAB classification, and CALGB category, with survival probabilities for 1, 3, and 5 years. **B,** CI illustrating the comparison between the nomogram-predicted overall survival probability and the actual overall survival probability.

We established and meticulously validated a predictive nomogram tailored for predicting outcomes in AML patients. This nomogram, presented in **Fig 7A**, integrates our microenvironment prognostic model, patient age, FAB classification, and CALGB category, providing risk assessments for patients at 1, 3, and 5-year intervals. Its development aimed to facilitate personalized risk evaluation and inform clinical decision-making. To gauge its performance in distinguishing patients who experienced the targeted clinical event from those who did, we employed the concordance index (C-index) (**Fig 7B**).

### 3.7 MPM’s Prognostic Accuracy in AML Clinical Context

**Fig 8:**
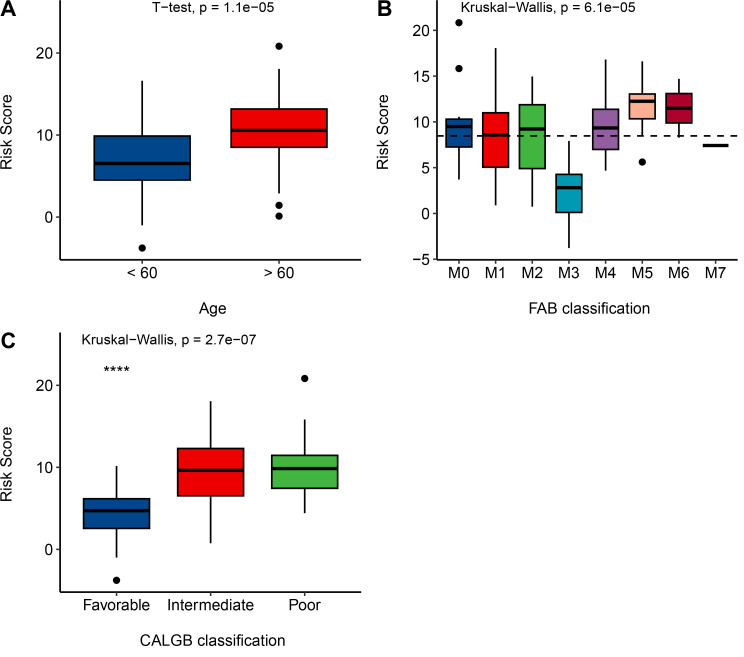
Validation of the MPM in Clinical Characteristics of AML Patients. **A**, MPM significantly correlates with AML patient age, distinguishing those under and above 60 years. (*p-value* = 1.1e-05). **B**, MPM shows a significant relationship with FAB classification, aiding in subtype-specific survival predictions (P = 6.1e-06). **C**, MPM is notably associated with cytogenetic status (CALGB criteria), offering precise risk stratification for favorable, intermediate, and poor cytogenetic patients (*p-value* = 2.7e-07).

Our analysis has uncovered a robust connection between the MPM and critical clinical parameters in AML patients, which include age, FAB classification, and cytogenetic status. These findings underscore the potential value of the MPM in customizing treatment approaches and enhancing patient care. The MPM has demonstrated significant associations with AML patient age, effectively distinguishing between patients below and above the age of 60 (*p-value* = 1.1e-05, **Fig 8A**). Furthermore, it exhibits a substantial correlation with the FAB (French-American-British) classification system, allowing for subtype-specific survival predictions (*p-value* = 6.1e-06, **Fig 8B**). Additionally, as illustrated in **Fig 8C**, the MPM has revealed notable relationships with cytogenetic status as characterized by CALGB criteria, enabling precise risk stratification for patients with favorable, intermediate, and poor cytogenetic profiles (*p-value* = 2.7e-07).

## 4 Discussion

AML represents the most common acute leukemia type in adults, characterized by a high mortality rate and variable outcomes [36]. Despite ongoing advancements in identifying new drugs and therapeutic targets, the five-year survival rate remains disappointingly low [37,38]. Accurately determining prognosis at diagnosis is crucial for improving overall survival rates in AML patients [39,40]. AML prognosis is influenced by several factors, including genetic abnormalities; however, the role of the bone marrow microenvironment has garnered significant attention in recent years. The bone marrow serves as the primary site for leukemia’s onset and progression, where stromal and immune cells within this microenvironment are pivotal in the proliferation, survival, and drug resistance of leukemic cells [41,42]. Our study delves into the intricate relationships between specific TME cell populations and AML prognosis, pioneering a MPM rooted in the ESTIMATE score to heighten the precision of prognostic biomarkers for AML. This exploration aims to uncover how the TME dictates the destiny of AML, paving the way for innovative therapeutic targets.

Using the ESTIMATE algorithm, we calculated the purity of microenvironment cells in the TME of AML patients. By comparing ESTIMATE scores among patients, which were highly correlated with FAB classification and overall survival, we classified our patients into two subgroups: high and low ESTIMATE scores. Subsequently, we identified differential expression genes (DEGs). Pathway enrichment analysis of TME-related DEGs revealed that these genes are remarkably associated with immune response, inflammatory response, and innate immune response pathways. Inflammation-related genes are attendant to AML progression and chemoresistance; therefore, the inflammatory response is known as a prognostic factor in these patients [43]. Also, there has been a growing number of studies involving immune-related processes as a prognostic factor in AML [44].

In our analysis, survival studies on DEGs facilitated the creation of the MPM. Notably, the overexpression of eight genes (CXCL12, GZMB, ITPR2, LYN, RAB9B, RGMB, RUFY4, TRIM16) correlated strongly with patient survival. Among these, the chemokine CXCL12 and its receptor CXCR4 are crucial for mediating interactions between leukemia cells and their microenvironment, promoting cell migration and survival, thereby contributing to chemotherapy resistance [45]. AML cells often express the CXCR4 receptor, which is the receptor for CXCL12. The interaction between CXCL12 and CXCR4 promotes the migration and homing of AML cells to the bone marrow microenvironment, where they can receive support and protection from the surrounding stromal cells. This interaction also contributes to the resistance of AML cells to chemotherapy, as CXCL12 signaling can activate survival pathways in the leukemic cells. Elevated CXCL12 levels in the bone marrow are invariably associated with adverse AML patient outcomes [46,47]. Granzyme B (GzmB), a serine protease from cytotoxic lymphocytes, targets and destroys virus-infected or malignant cells [48]. In AML, however, the levels of GZMB are notably diminished, undermining its role as a crucial cytotoxic mediator for T and NK cells in combatting cancer cells [49]. The ITPR2 gene encodes for the Inositol 1,4,5-trisphosphate receptor type 2 (IP3R2) protein, a critical calcium channel that modulates intracellular signaling pathways. Although initial studies suggest a prognostic role for ITPR2, its implications in AML remain underexplored [50,51]. LYN, a tyrosine kinase, is integral to internal signaling processes and is crucial for the differentiation and persistence of the leukemic phenotype across various blood cancers including AML, CML, and B-cell lymphocytic leukemia [52]. Research has indicated a significant association between LYN activity and overall survival in AML [53]. RAB9B, belonging to the RAB family of GTPases, is essential for vesicle trafficking and protein transport within cells. While there is no study on RGMB in AML, Jiang et.al found that RAB9B is overexpressed in colorectal cancer and promotes tumor growth and metastasis [54]. RGMB, a member of the repulsive guidance molecule family, is known for its role in neuronal development and axon guidance. RGMB is overexpressed in glioblastoma and promotes tumor growth and invasion. in terms of tumor progression, RGMb can inhibit cell proliferation, and invasion, in NSCLC, breast cancer, liver cancer, squamous cell carcinoma, nasopharyngeal carcinoma and other related tumors, so as to inhibit tumor progression and even improving survival ratio [55]. RUFY4 (RUN and FYVE domain-containing protein 4) is a protein that plays a role in intracellular membrane trafficking and cytoskeletal organization [56]. the role of RUFY4 is not well understood in AML. However, studies have shown its involvement in regulating autophagy, a cellular process that plays a role in cancer development and progression [57]. TRIM16 (Tripartite Motif Containing 16), is a member of the tripartite motif (TRIM) family of proteins [58]. While there is limited research on TRIM16 in AML, studies have shown its involvement in other types of cancers and cellular processes. Previous studies demonstrated that TRIM16 is involved in regulating the NF-κB signaling pathway, which plays a crucial role in cancer development and progression [59].

We developed an integrated prognostic model combining univariate Cox regression, LASSO regression, and multivariate Cox regression, augmented by Kaplan-Meier survival curves and nomograms. This model, leveraging genes such as CXCL12, GZMB, ITPR2, LYN, RAB9B, RGMB, RUFY4, and TRIM16, was designed to forecast outcomes for AML patients. The efficacy of this model was corroborated through ROC curve analysis across training, testing, and overall patient groups. It demonstrated a notably high AUC in the TARGET dataset, and the Kaplan-Meier analysis also yielded significant results. The findings revealed that patients in the high-risk category exhibited markedly lower survival rates compared to those in the low-risk group. Moreover, the model’s prognostic relevance was significantly associated with patient age, the FAB classification, and the CALGB classification.

Nonetheless, this study presents certain limitations. First, the prognostic model was constructed from retrospective data, necessitating prospective multi-institutional cohort studies to validate its clinical applicability and reproducibility across heterogeneous populations. While external validation was performed using the TARGET database, broader evaluation across geographically and ethnically diverse cohorts is essential to confirm the model’s generalizability, particularly given potential variability in tumor microenvironment composition influenced by genetic ancestry or regional therapeutic practices. Second, the reliance on bulk RNA sequencing and computational deconvolution algorithms such as ESTIMATE introduces inherent technical constraints, including limited resolution to dissect cellular heterogeneity and potential batch effects across sequencing platforms. Although emerging single-cell transcriptomic and spatially resolved omics technologies could address these limitations by providing high-resolution microenvironmental mapping, their integration into clinical workflows remains constrained by infrastructural requirements, technical expertise, and standardization challenges—critical barriers for resource-limited settings. Third, while the model demonstrates robust prognostic stratification, its translational utility requires rigorous validation through prospective clinical trials assessing its capacity to guide therapeutic decisions, particularly in the context of emerging targeted therapies against TME components. Despite these considerations, our predictive framework provides a promising foundation for identifying novel therapeutic targets, ultimately informing more robust diagnostic and treatment paradigms for AML.

## 5 Conclusion

Our study establishes a Tumor Microenvironment-derived Prognostic Model (MPM) that integrates eight TME-associated genes (CXCL12, GZMB, ITPR2, LYN, RAB9B, RGMB, RUFY4, TRIM16) to stratify AML patients into distinct risk categories with significant survival differences. Unlike existing models that focus on genetic mutations or immunogenic cell death markers, the MPM uniquely leverages stromal and immune infiltration metrics derived from the ESTIMATE algorithm, capturing dynamic interactions between leukemic cells and their protective niche. The model demonstrated robust prognostic accuracy across two independent cohorts (TCGA and TARGET), outperforming conventional parameters such as age and cytogenetic risk. Clinically, the MPM offers a actionable framework for risk-adapted therapy: high-risk patients could be prioritized for intensive regimens or novel TME-targeted therapies (e.g., CXCR4 inhibitors), while low-risk patients might benefit from reduced-intensity protocols to minimize toxicity. Future steps include prospective validation in multicenter trials to assess its utility in guiding real-time therapeutic decisions and integration into digital platforms for rapid risk scoring, thereby bridging the gap between computational biology and bedside practice.

## Supporting information

S1 Fig Comparative ROC Curve analysis for 1, 3, and 5-year prognostic accuracy. A, ESTIMATE algorithm; B, patient age in AML cohort.
(PDF)

## References

[1] Mizani S, Keshavarz A, Vazifeh Shiran N, Bashash D, Allahbakhshian Farsani F. Expression changes of SIRT1 and FOXO3a significantly correlate with oxidative stress resistance genes in AML patients. Indian Journal of Hematology and Blood Transfusion. 2022;1–10. 10.1007/s12288-022-01612-3 PMC10247606 37304466

[2] Bakhtiyari M, Liaghat M, Aziziyan F, Shapourian H, Yahyazadeh S, Alipour M, et al. The role of bone marrow microenvironment (BMM) cells in acute myeloid leukemia (AML) progression: immune checkpoints, metabolic checkpoints, and signaling pathways. Cell Commun Signal. 2023;21(1):252. doi: 10.1186/s12964-023-01282-2 37735675 PMC10512514

[3] Cheung HL, Wong YH, Li YY, Yang X, Ko LH, Tan Kabigting JE, et al. Microenvironment matters: In vitro 3D bone marrow niches differentially modulate survival, phenotype and drug responses of acute myeloid leukemia (AML) cells. Biomaterials. 2025;312:122719. doi: 10.1016/j.biomaterials.2024.122719 39088912

[4] Lisi-Vega LE, Pievani A, García-Fernández M, Forte D, Williams TL, Serafini M, et al. Bone marrow mesenchymal stromal cells support translation in refractory acute myeloid leukemia. Cell Rep. 2025;44(1):115151. doi: 10.1016/j.celrep.2024.115151 39932190 PMC7617453

[5] Balkwill FR, Capasso M, Hagemann T. The tumor microenvironment at a glance. J Cell Sci. 2012;125(Pt 23):5591–6. doi: 10.1242/jcs.116392 23420197

[6] Bejarano L, Jordāo MJC, Joyce JA. Therapeutic Targeting of the Tumor Microenvironment. Cancer Discov. 2021;11(4):933–59. doi: 10.1158/2159-8290.CD-20-1808 33811125

[7] Hajipirloo LK, Nabigol M, Khayami R, Karami N, Farsani MA, Navidinia AA. Construction of a stromal-related prognostic model in acute myeloid leukemia by comprehensive bioinformatics analysis. Curr Res Transl Med. 2025;73(2):103492. doi: 10.1016/j.retram.2025.103492 39818173

[8] Feng D-C, Zhu W-Z, Wang J, Li D-X, Shi X, Xiong Q, et al. The implications of single-cell RNA-seq analysis in prostate cancer: unraveling tumor heterogeneity, therapeutic implications and pathways towards personalized therapy. Mil Med Res. 2024;11(1):21. doi: 10.1186/s40779-024-00526-7 38605399 PMC11007901

[9] Tettamanti S, Pievani A, Biondi A, Dotti G, Serafini M. Catch me if you can: how AML and its niche escape immunotherapy. Leukemia. 2022;36(1):13–22. doi: 10.1038/s41375-021-01350-x 34302116 PMC8727297

[10] Ciantra Z, Paraskevopoulou V, Aifantis I. The rewired immune microenvironment in leukemia. Nature Immunology. 2025;1–15. 40021898 10.1038/s41590-025-02096-9

[11] Goda C, Kulkarni R, Bustos Y, Li W, Rudich A, Balcioglu O, et al. Cellular taxonomy of the preleukemic bone marrow niche of acute myeloid leukemia. Leukemia. 2025;39(1):51–63. doi: 10.1038/s41375-024-02415-3 39358541 PMC11717697

[12] Duarte D, Hawkins ED, Lo Celso C. The interplay of leukemia cells and the bone marrow microenvironment. Blood. 2018;131(14):1507–11. doi: 10.1182/blood-2017-12-784132 29487069

[13] Korn C, Méndez-Ferrer S. Myeloid malignancies and the microenvironment. Blood. 2017;129(7):811–22. doi: 10.1182/blood-2016-09-670224 28064238 PMC5314811

[14] Zeng Z, Shi YX, Samudio IJ, Wang R-Y, Ling X, Frolova O, et al. Targeting the leukemia microenvironment by CXCR4 inhibition overcomes resistance to kinase inhibitors and chemotherapy in AML. Blood. 2009;113(24):6215–24. doi: 10.1182/blood-2008-05-158311 18955566 PMC2699240

[15] Pitt LA, Tikhonova AN, Hu H, Trimarchi T, King B, Gong Y. CXCL12-producing vascular endothelial niches control acute T cell leukemia maintenance. Cancer Cell. 2015;27(6):755–68. 26058075 10.1016/j.ccell.2015.05.002 PMC4461838

[16] Zhong Y, Xu F, Wu J, Schubert J, Li MM. Application of Next Generation Sequencing in Laboratory Medicine. Ann Lab Med. 2021;41(1):25–43. doi: 10.3343/alm.2021.41.1.25 32829577 PMC7443516

[17] Zhou R, Jia X, Li Z, Huang S, Feng W, Zhu X. Identifying an immunosenescence-associated gene signature in gastric cancer by integrating bulk and single-cell sequencing data. Sci Rep. 2024;14(1):17055. doi: 10.1038/s41598-024-68054-x 39048596 PMC11269723

[18] Wang X, Ye Z, Zhou L, Chen Y. Clinical and Prognostic Implications of an Alternative Splicing-related Risk Model Based on TP53 Status in Breast Cancer. Curr Pharm Biotechnol. 2025;26(2):246–59. doi: 10.2174/0113892010283176240212073417 39916426

[19] Sun W, Shi H, Yuan Z, Xia L, Xiang X, Quan X, et al. Prognostic Value of Genes and Immune Infiltration in Prostate Tumor Microenvironment. Front Oncol. 2020;10:584055. doi: 10.3389/fonc.2020.584055 33194726 PMC7662134

[20] Wang Y, Wang Y, Xu C, Liu Y, Huang Z. Identification of Novel Tumor-Microenvironment-Regulating Factor That Facilitates Tumor Immune Infiltration in Colon Cancer. Mol Ther Nucleic Acids. 2020;22:236–50. doi: 10.1016/j.omtn.2020.08.029 33230430 PMC7515980

[21] Gao Z, Chen S, Ye W. Cuproptosis related lncRNA signature as a prognostic and therapeutic biomarker in osteosarcoma immunity. Sci Rep. 2025;15(1):221. doi: 10.1038/s41598-024-84024-9 39747262 PMC11696132

[22] Luo J, Xie Y, Zheng Y, Wang C, Qi F, Hu J, et al. Comprehensive insights on pivotal prognostic signature involved in clear cell renal cell carcinoma microenvironment using the ESTIMATE algorithm. Cancer Med. 2020;9(12):4310–23. doi: 10.1002/cam4.2983 32311223 PMC7300420

[23] Xiang S, Li J, Shen J, Zhao Y, Wu X, Li M, et al. Identification of Prognostic Genes in the Tumor Microenvironment of Hepatocellular Carcinoma. Front Immunol. 2021;12:653836. doi: 10.3389/fimmu.2021.653836 33897701 PMC8059369

[24] Liu Z, Huang X. A model based on eight iron metabolism-related genes accurately predicts acute myeloid leukemia prognosis. Biocell. 2023;47(3):593–605. doi: 10.32604/biocell.2023.024148

[25] Xie J-Y, Wang W-J, Wang N, Dong Q, Han H, Feng Y-P, et al. A novel immune-related gene signature correlated with serum IL33 expression in acute myeloid leukemia prognosis. Am J Transl Res. 2023;15(6):4332–44. 37434810 PMC10331686

[26] Zhong F, Yao F, Cheng Y, Liu J, Zhang N, Li S, et al. m6A-related lncRNAs predict prognosis and indicate immune microenvironment in acute myeloid leukemia. Sci Rep. 2022;12(1):1759. doi: 10.1038/s41598-022-05797-5 35110624 PMC8810799

[27] Ni J, Wu Y, Qi F, Li X, Yu S, Liu S, et al. Screening the Cancer Genome Atlas Database for Genes of Prognostic Value in Acute Myeloid Leukemia. Front Oncol. 2020;9:1509. doi: 10.3389/fonc.2019.01509 32039005 PMC6990132

[28] Hajipirloo LK, Nabigol M, Khayami R, Karami N, Farsani MA, Navidinia AA. Construction of a stromal-related prognostic model in acute myeloid leukemia by comprehensive bioinformatics analysis. Curr Res Transl Med. 2025;73(2):103492. doi: 10.1016/j.retram.2025.103492 39818173

[29] Peroni E, Randi ML, Rosato A, Cagnin S. Acute myeloid leukemia: from NGS, through scRNA-seq, to CAR-T. dissect cancer heterogeneity and tailor the treatment. J Exp Clin Cancer Res. 2023;42(1):259. doi: 10.1186/s13046-023-02841-8 37803464 PMC10557350

[30] Wang M, Lindberg J, Klevebring D, Nilsson C, Lehmann S, Grönberg H, et al. Development and Validation of a Novel RNA Sequencing-Based Prognostic Score for Acute Myeloid Leukemia. J Natl Cancer Inst. 2018;110(10):1094–101. doi: 10.1093/jnci/djy021 29506270 PMC6186516

[31] Ng SWK, Mitchell A, Kennedy JA, Chen WC, McLeod J, Ibrahimova N, et al. A 17-gene stemness score for rapid determination of risk in acute leukaemia. Nature. 2016;540(7633):433–7. doi: 10.1038/nature20598 27926740

[32] Chuang M-K, Chiu Y-C, Chou W-C, Hou H-A, Chuang EY, Tien H-F. A 3-microRNA scoring system for prognostication in de novo acute myeloid leukemia patients. Leukemia. 2015;29(5):1051–9. doi: 10.1038/leu.2014.333 25428263

[33] Sheng X, Lu J, Wang J, Fan K, Huang M, Lu Q. Construction of a prognostic model of acute myeloid leukemia associated with immunogenic cell death. Expert Review of Hematology. 2023;1–9. 10.1080/17474086.2023.2208861 37114857

[34] Chen M, Zeng Z, Qin W, Cai X, Lu X, Chen S. A novel prognostic model of methylation-associated genes in acute myeloid leukemia. Clinical and Translational Oncology. 2023;1–10. 10.1007/s12094-022-03069-2 PMC10203004 36715873

[35] Zeng T, Cui L, Huang W, Liu Y, Si C, Qian T, et al. The establishment of a prognostic scoring model based on the new tumor immune microenvironment classification in acute myeloid leukemia. BMC Med. 2021;19(1):176. doi: 10.1186/s12916-021-02047-9 34348737 PMC8340489

[36] Bastola S, Rai M, Ojbindra K. Trends in acute myeloid leukemia (AML) hospitalization: incidence, mortality, length of stay, and financial burden from 2011 to 2018. Blood. 2024;144:2872.

[37] Zare R, Haghshenas H, Moghaddam S, Avazpour R, Kargar Jahromi H, Delam H. The Survival Rate of Leukemia Patients in Asian Regions: A Systematic Review and Meta-Analysis Study. Iranian Journal of Pediatric Hematology and Oncology. 2025;15(1):386–410.

[38] Han HJ, Choi K, Suh HS. Impact of aging on acute myeloid leukemia epidemiology and survival outcomes: A real-world, population-based longitudinal cohort study. PLoS One. 2024;19(5):e0300637. doi: 10.1371/journal.pone.0300637 38771863 PMC11108202

[39] Devarkonda V, Akabane H. Transforming acute myeloid leukemia treatment through next-generation sequencing: a single-center experience. Cureus. 2023;15(9). 10.7759/cureus.45917 PMC10599270 37885525

[40] Dong L, Dai G, Zhao J. Impact of body mass index at diagnosis on outcomes of pediatric acute leukemia: A systematic review and meta-analysis. PLoS One. 2024;19(5):e0302879. doi: 10.1371/journal.pone.0302879 38709714 PMC11073705

[41] Hino C, Pham B, Park D, Yang C, Nguyen MHK, Kaur S, et al. Targeting the Tumor Microenvironment in Acute Myeloid Leukemia: The Future of Immunotherapy and Natural Products. Biomedicines. 2022;10(6):1410. doi: 10.3390/biomedicines10061410 35740430 PMC9219790

[42] Hu F, Wang Y, Wang W, Gale RP, Wu B-Y, Liang Y. Improving prediction accuracy in acute myeloid leukaemia: micro-environment, immune and metabolic models. Leukemia. 2021;35(11):3073–7. doi: 10.1038/s41375-021-01377-0 34365474 PMC8550966

[43] Puissant A, Medyouf H. Walking the Tightrope: Balancing Delicate Inflammation Response to Eradicate Acute Myeloid Leukemia. Cancer Discov. 2022;12(7):1617–9. doi: 10.1158/2159-8290.CD-22-0473 35791696

[44] Li B, Li T, Zheng X, Zu X, Yin Q, Qin L. The potential novel immune-related prognostic factors for acute myeloid leukemia. 2022.

[45] Wang S-S, Xu Z-J, Jin Y, Ma J-C, Xia P-H, Wen X, et al. Clinical and prognostic relevance of CXCL12 expression in acute myeloid leukemia. PeerJ. 2021;9:e11820. doi: 10.7717/peerj.11820 34327063 PMC8300536

[46] Yazdani Z, Mousavi Z, Moradabadi A, Hassanshahi G. Significance of CXCL12/CXCR4 Ligand/Receptor Axis in Various Aspects of Acute Myeloid Leukemia. Cancer Manag Res. 2020;12:2155–65. doi: 10.2147/CMAR.S234883 32273755 PMC7102884

[47] Ladikou EE, Chevassut T, Pepper CJ, Pepper AG. Dissecting the role of the CXCL12/CXCR4 axis in acute myeloid leukaemia. Br J Haematol. 2020;189(5):815–25. doi: 10.1111/bjh.16456 32135579

[48] Wang H, Huang Y, He J, Zhong L, Zhao Y. Dual roles of granzyme B. Scandinavian Journal of Immunology. 2021;94(3):e13086.

[49] Kamarudin N, Ismail I, Hassan R, Johan MF, Ramli M. Gene expression profiling in normal cytogenetic acute myeloid leukaemia of Malay patient. Bangladesh J Med Sci. 2021;20(3):556–62. doi: 10.3329/bjms.v20i3.52798

[50] Zheng S, Wang X, Zhao D, Liu H, Hu Y. Calcium homeostasis and cancer: insights from endoplasmic reticulum-centered organelle communications. Trends Cell Biol. 2023;33(4):312–23. doi: 10.1016/j.tcb.2022.07.004 35915027

[51] Zhai X, Chen X, Wan Z, Ge M, Ding Y, Gu J, et al. Identification of the novel therapeutic targets and biomarkers associated of prostate cancer with cancer-associated fibroblasts (CAFs). Front Oncol. 2023;13:1136835. doi: 10.3389/fonc.2023.1136835 36937411 PMC10020494

[52] Heo S-K, Noh E-K, Yoon D-J, Jo J-C, Choi Y, Koh S, et al. Radotinib Induces Apoptosis of CD11b+ Cells Differentiated from Acute Myeloid Leukemia Cells. PLoS One. 2015;10(6):e0129853. doi: 10.1371/journal.pone.0129853 26065685 PMC4466365

[53] Patel RK, Weir MC, Shen K, Snyder D, Cooper VS, Smithgall TE. Expression of myeloid Src-family kinases is associated with poor prognosis in AML and influences Flt3-ITD kinase inhibitor acquired resistance. PLoS One. 2019;14(12):e0225887. doi: 10.1371/journal.pone.0225887 31790499 PMC6886798

[54] Jiang X, Yang L, Gao Q, Liu Y, Feng X, Ye S, et al. The Role of RAB GTPases and Its Potential in Predicting Immunotherapy Response and Prognosis in Colorectal Cancer. Front Genet. 2022;13:828373. doi: 10.3389/fgene.2022.828373 35154286 PMC8833848

[55] Li S, Zhang R, Zhang L, Qin X. Research Progress on RGMb and its Signaling Pathway. IJBLS. 2023;2(3):39–43. doi: 10.54097/ijbls.v2i3.8650

[56] Char R, Pierre P. The RUFYs, a Family of Effector Proteins Involved in Intracellular Trafficking and Cytoskeleton Dynamics. Front Cell Dev Biol. 2020;8:779. doi: 10.3389/fcell.2020.00779 32850870 PMC7431699

[57] Pesce M, Ballerini P, Paolucci T, Puca I, Farzaei MH, Patruno A. Irisin and Autophagy: First Update. Int J Mol Sci. 2020;21(20):7587. doi: 10.3390/ijms21207587 33066678 PMC7588919

[58] Azuma K, Inoue S. Efp/TRIM25 and Its Related Protein, TRIM47, in Hormone-Dependent Cancers. Cells. 2022;11(15):2464. doi: 10.3390/cells11152464 35954308 PMC9368238

[59] Zhou B, Huang Y, Feng Q, Zhu H, Xu Z, Chen L, et al. TRIM16 promotes aerobic glycolysis and pancreatic cancer metastasis by modulating the NIK-SIX1 axis in a ligase-independent manner. Am J Cancer Res. 2022;12(11):5205–25. 36504902 PMC9729885

