# Supplementary figures and images for: Bioinformatics-guided construction of a tumor microenvironment-derived prognostic model in acute myeloid leukemia
Source: PLoS One. 2025 Jul 3;20(7):e0325145. doi: 10.1371/journal.pone.0325145 (PMC12225840; doi:10.1371/journal.pone.0325145)

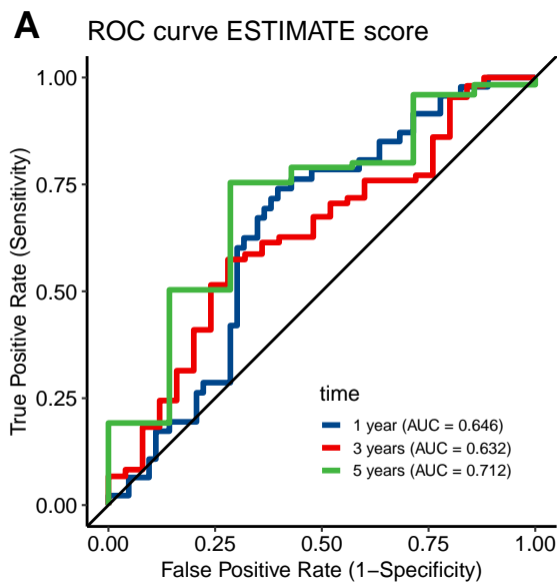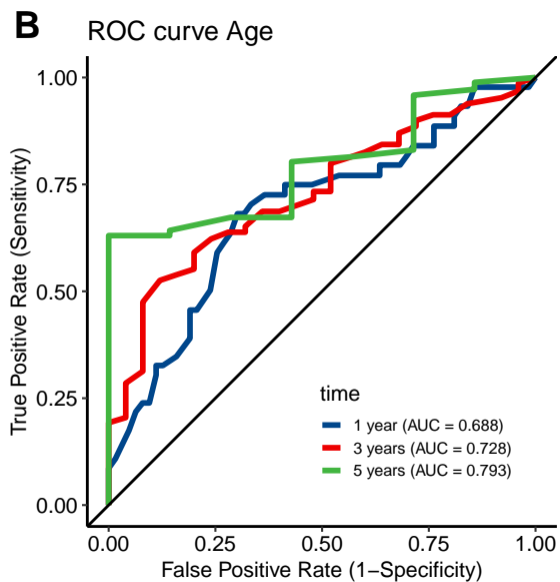

Supplement: S1 Fig — A, ESTIMATE algorithm; B, patient age in AML cohort. (PDF) [file pone.0325145.s001.pdf]
